# Increased Clot Formation in the Absence of Increased Thrombin Generation in Patients with Peripheral Arterial Disease: A Case–Control Study

**DOI:** 10.3389/fcvm.2017.00023

**Published:** 2017-04-20

**Authors:** Marie-Claire F. Kleinegris, Joke Konings, Jan W. Daemen, Yvonne Henskens, Bas de Laat, Henri M. H. Spronk, Arina J. ten Cate-Hoek, Hugo ten Cate

**Affiliations:** ^1^Laboratory for Clinical Thrombosis and Hemostasis, Department of Internal Medicine, Cardiovascular Research Institute Maastricht, Maastricht University Medical Center, Maastricht, Netherlands; ^2^Department of Biochemistry, Cardiovascular Research Institute Maastricht, Maastricht University Medical Centre, Maastricht, Netherlands; ^3^Synapse BV, Maastricht, Netherlands; ^4^Department of Surgery, Maastricht University Medical Center, Maastricht, Netherlands; ^5^Central Diagnostic Laboratory, Maastricht University Medical Centre, Maastricht, Netherlands

**Keywords:** coagulation, intermittent claudication, peripheral arterial disease, thrombin generation, thromboelastometry

## Abstract

**Background:**

In peripheral arterial disease (PAD), activation of the hemostatic system may contribute to atherosclerosis progression and atherothrombotic events.

**Objective:**

This case–control study assesses the overall coagulation status in PAD patients by evaluating coagulation markers in combination with thrombin generation potential, whole blood (WB) clot formation, and fibrinolysis.

**Methods:**

In blood from 40 PAD patients (*n* = 20 with cardiovascular event within 1 year after initial diagnosis, *n* = 20 without) and 40 apparently healthy controls, thrombin generation was determined in WB and platelet-poor plasma. Whole blood rotational thromboelastometry (ROTEM) measurements were triggered with tissue factor with/without tissue plasminogen activator.

**Results:**

We observed increased levels of erythrocyte sedimentation rate, leukocytes, eosinophil granulocytes, vWF antigen, fibrinogen, and D-dimer in PAD patients (*p* < 0.05). Markers of thrombin generation potential showed no difference between patients and healthy controls. In PAD patients with event compared to patients without, WB-thrombin generation showed a lower thrombin potential when triggered with 0 and 2.5 pM tissue factor. The ROTEM clotting assay showed significantly faster clot formation and increased clot firmness in PAD patients compared to controls. No significant differences were found for parameters of clot degradation.

**Conclusion:**

There are no significant differences between the thrombin generation profiles of PAD patients and healthy controls. Between PAD patients with and without cardiovascular event, the WB thrombin generation appears to differ. Mechanistically, PAD patients show an increased ability to form a stable clot in WB in comparison to healthy controls. This is most likely due to the increased fibrinogen levels related to the inflammation in atherosclerosis, confirming the importance of the inflammation-coagulation axis.

## Introduction

Peripheral arterial disease (PAD) results from atherosclerosis in the major arteries of the lower extremities, causing an imbalance between the demand and supply of oxygenated blood flow (1). This decreased blood flow in the legs can cause “intermittent claudication,” characterized by a cramp-like pain or aching in the calves or thighs typically brought on by ambulation and relieved with rest (2). The total disease prevalence of PAD is estimated to be 2–7% in the general population aged 50–70 years and believed to increase to 15–20% in people older than 70 years (3). Due to this high prevalence, PAD should be considered an important global healthcare problem, in particular, since patients with PAD have a high risk of cardiovascular complications and death (2). After initial diagnosis, morbidity and mortality rates after 5, 10, and 15 years are approximately 30, 50, and 70%, respectively. Several studies suggest that ongoing activation of the hemostatic system may contribute to both atherosclerotic progression and the occurrence of atherothrombotic events (4). Indeed, in a recent meta-analysis, we showed that an increased D-dimer was independently associated with a twofold increased risk of cardiovascular events in the near-term future (within 4 years of the assessment of D-dimer) (5). Previously it was demonstrated that PAD patients have a systemic increase in concentrations of various rheological and hemostatic factors. These factors include a significant increase of fibrinogen, thrombin–antithrombin complexes, prothrombin fragment 1.2 (F1.2), and D-dimer (6–12). In this study, we hypothesize that patients with PAD show a hypercoagulable and activated inflammatory state that can be observed in both plasmatic coagulation as in whole blood (WB) clot formation. We therefore wanted to gain insight into the *overall* hemostatic profile (i.e., coagulation, clot formation, and clot lysis capacity) of patients with occlusive PAD versus that of healthy controls. Furthermore, we wanted to assess whether PAD patients who experienced a cardiovascular event within 1 year of follow-up after the initial diagnosis of PAD differed in their hemostatic profile from PAD patients who did not experience a cardiovascular event. This based on the hypothesis that a hypercoagulable state in PAD patients leads to more atherothrombotic events.

Therefore, we performed a nested case–control study in which we assessed the levels of various coagulation markers, both the plasmatic thrombin generation potential with the calibrated automated thrombogram (CAT) and the recent WB thrombin generation analysis. In addition, we studied the fibrin formation and lysis effects by a modified tissue factor-triggered rotational thromboelastometry (ROTEM) test in WB.

## Patients and Methods

### Patients

From 2009 to 2013, 301 consecutive patients from the vascular surgery departments of 3 hospitals in South-Limburg, the Netherlands, with newly diagnosed occlusive PAD were enrolled in a prospective cohort study with a follow-up duration of 2 years. The diagnosis of occlusive PAD was based on an ankle-brachial index <0.9. After 1-year follow-up in this study, patients with one or more cardiovascular events during this period were selected for this nested case–control study as “cases.” From this cohort, a total number of 20 cases were thus identified. Patients, from the same cohort, with PAD and of comparable age and sex but without CV event during this follow-up were selected as “controls” (*n* = 20). The study was set up as an explorative, hypothesis-generating study to obtain insights into the prothrombotic mechanism in PAD patients; therefore, a formal sample size calculation was not included. Cardiovascular events were defined as ischemic cerebrovascular accident (CVA), transient ischemic attack (TIA), new unstable angina pectoris, myocardial infarction (MI), coronary revascularization, and/or revascularization for PAD. Exclusion criteria were the use of medication known to affect coagulation (e.g., coumadins, direct factor Xa or thrombin inhibitors, or heparin), documented congenital coagulation disorders, documented chronic inflammatory disease (however, COPD Gold I and II were accepted), active malignancy, current pregnancy, and age <18 years. Treatment with platelet aggregation inhibitors, as is the current standard of care for PAV, was allowed in this study design.

### Healthy Subjects

Forty apparently healthy individuals of comparable sex and age (mean age of 67 years, 55% males) were also enrolled in the study, after informed consent was obtained. The Edinburgh Claudication Questionnaire (ECQ) was used to select controls in which the presence of PAD was deemed unlikely. Exclusion criteria were the use of medication known to affect coagulation (e.g., coumadins, direct factor Xa or thrombin inhibitors, or heparin), known congenital coagulation disorders, chronic inflammatory disease, active malignancy, current pregnancy, and age <18 years.

### Normal Pooled Plasma

Normal pooled plasma (stored frozen at −80°C) used for the normalization of thrombin generation samples was prepared by pooling plasma from 80 to 90 apparently healthy volunteers.

### Blood Collection and Plasma Preparation

Venous blood was collected in a 4.0-mL EDTA tube [Vacutainer plastic, K2E (EDTA) 7.2 mg, Becton and Dickinson] and 4.5 mL 3.2% citrate tubes (Vacutainer glass, 0.105 M, Becton and Dickinson). Blood was drawn by clean venipuncture in the antecubital fossa and within 1 h processed to obtain platelet-poor plasma (PPP). PPP was obtained by initial centrifugation at 2,500 *g* for 5 min (18°C) followed by centrifugation at 10,000 *g* for 10 min (18°C) and stored at −80°C until use. All samples were thawed for 15 min at 37°C before testing. One 4.5 mL 3.2% citrate tube (Vacutainer glass, 0.105 M, Becton and Dickinson) was used to collect WB for the “tPA-ROTEM.”

### Measurements

#### Conventional Coagulation Tests and Hematologic Parameters

Hemoglobin, hematocrit, erythrocyte sedimentation rate (ESR), thrombocyte count, leukocyte count and leukocyte differentiation, red blood cell distribution width, and mean platelet volume measurements were performed on a Sysmex XN-9000 (Sysmex Corporation, Kobe, Japan) using blood from an EDTA tube. The measurements of activated partial thromboplastin time (Dade^®^ Actin^®^ FSL Activated PTT Reagent, Calcium Chloride Solution), PT (Dade^®^Innovin^®^), fibrinogen (Dade^®^ Thrombin Reagent), D-dimer (INNOVANCE D-Dimer), and vWF antigen were performed on the Sysmex CS-2100i analyzer (Sysmex Corporation, Kobe, Japan) according to the manufacturer’s instructions (all reagents by Siemens Healthcare Diagnostics Products, Marburg, Germany).

#### Plasma Thrombin Generation

Thrombin generation in tissue factor-triggered PPP was measured by means of the CAT method (Thrombinoscope BV, Maastricht, the Netherlands) according to our in-house standardized procedure (13). In brief, measurements were conducted on 80 µL PPP with a final concentration of 1 or 5 pM tissue factor (PPP reagent, PPP reagent low; thrombinoscope BV) and in the presence of 4 µM phospholipids. The measurements were conducted in the absence or presence of 0.56 nM recombinant soluble thrombomodulin (Thrombinoscope).

#### WB Thrombin Generation

Whole blood thrombin generation was performed as previously described with minor modifications (14). Briefly, WB (50%) was mixed with a rhodamine-based thrombin substrate (300 µM; P2Rho, Diagnostica Stago Gennevilliers, France) and activated with a mixture of tissue factor (0, 0.5, or 2.5 pM) and CaCl_2_ (16.7 mM). For calibration, 2-macroglobulin-thrombin complex (100 nM thrombin activity; α2M-T) was added to the mixture of WB and rhodamine-based thrombin substrate (all concentrations are final concentrations). After activation, the mixture was transferred onto 6-mm paper disks (Whatman 589/1, Whatman GmbH) and covered with mineral oil (USB Corporation). Samples were run in triplicate for 50 min, and fluorescence was recorded every 6 s with a Fluoreskan Ascent multiplate fluorometer with λex = 485 nm and λem = 538 nm (Thermolabsystems, Helsinki, Finland). All procedures were performed at 37°C.

#### Rotational Thromboelastometry

The tissue factor-triggered ROTEM (EXTEM based) and the “tPA-ROTEM” give a graphic representation of the clot formation process, and due to the addition of tissue plasminogen activator (tPA) also include the subsequent fibrinolysis process. Both the ROTEM (EXTEM based) and the “tPA-ROTEM” were carried out by means of the four-channel ROTEM^®^ Gamma device operated according to manufacturer instructions using tissue factor (Innovin) and tPA (Actilyse). For the tPA-modified ROTEM procedure, recalcified citrated blood (300 µL) was incubated, within 1 h after venipuncture, at 37°C in a preheated cup. For the purpose of this study, the tPA-ROTEM was determined in recalcified citrated WB incubated with two different triggers: 35 pM tissue factor alone in the presence or absence of 175 ng/mL tPA (Kuiper et al., unpublished data). The following numerical parameters investigated on WB were (1) α-angle (α) defined as the speed (seconds per minute) at which the clot is formed; (2) clot formation time (CFT), defined as the time (seconds) from initiation of clotting until a clot firmness of 20 mm is detected; (3) maximum clot firmness (MCF) defined as the maximal amplitude (millimeters) of the clot; (4) lysis onset time (LOT) defined as the time (seconds) until 15% reduction of the MCF; (5) lysis time (LT) defined as the time (seconds) until 90% reduction of the MCF; and (6) fibrinolysis velocity (FV) defined as the decline in % per minute between the LOT and the LT.

Fibrinolysis capacity was assessed with the addition of 175 ng/mL recombinant tPA. This concentration was chosen after our lab determined that this was the optimal concentration to distinguish between a hypofibrinolytical or normofibrinolytical potential in ROTEM measurements with an acceptable runtime.

### Statistical Analyses

Statistical analysis was performed using the GraphPad Prism version 6.00 software (GraphPad Software, Inc., For Mac OS X, San Diego, CA, USA) and IBM SPSS Statistics 22 for Windows (IBM Corp., Armonk, NY, USA). Normality of the data was tested with the D’Agostino and Pearson omnibus normality test. According to the distribution of the variables, data are expressed as median ± interquartile ranges or mean ± SD. Differences between the results of the PAD patients with an event, PAD patients without an event, and the healthy controls groups were analyzed with the Kruskal–Wallis ANOVA with Dunn’s posttest or with the one-way ANOVA with Bonferroni multiple comparison test. The Mann–Whitney *U* test was used to test for differences between median values of two separate groups: all PAD patients and the healthy controls. A *p*-value of <0.05 was considered to be statistically significant.

The associations between the hemostatic markers, thrombin generation measurements, and the (tPA-) ROTEM were assessed using Spearman correlation coefficient. Statistical significance was considered as a two-tailed probability <0.05.

## Results

The main characteristics of the study population (*n* = 80) are reported in Table 1. Main differences between PAD patients and healthy controls are the history of tobacco use, the use of platelet aggregation inhibitors, the use of cholesterol lowering medication, and the prevalence of hypertension (all significantly higher in the PAD patients). Inspection of the individual clinical records showed that 17 PAD patients underwent a revascularization procedure for PAD and 3 patients had another ischemic cardiovascular event (1 patient experienced a retinal infarction during the revascularization procedure with 2 stents for renal artery stenosis, 1 patient experienced a TIA, and 1 patient had unstable angina with ischemia in multiple areas on thallium scintigraphy). These 20 events made up the cardiovascular events that classified PAD patients as “cases” (*n* = 20), and the other 20 PAD patients (“controls”) had remained free of cardiovascular events during the follow-up after the diagnosis of PAD.

**Table 1:** **Demographic and clinical characteristics of the study population (median with IQR)**.

Characteristics	PAD patients (*n* = 40)	PAD with event during first-year FU (*n* = 20)	PAD without event during first-year FU (*n* = 20)	Healthy controls (*n* = 40)	*p*-Value
**Demographics**					
Age (years)	67 (65–72)	67 (61–73)	68 (65–72)	67 (60–71)	0.2
Male sex (%)	24 (60)	9 (45)	15 (75)	22 (55)	0.8
BMI (kg/m^2^)	26.1 (23.7–29.3)	26.2 (23.5–29.3)	25.9 (24.0–29.8)	26.0 (23.4–27.8)	0.4
**Medical history**					
**Smoking (%)**					
Never smoked	1 (2.5)	1 (5)		16 (40.0)	<0.0001
Active smokers	15 (37.5)	8 (40)	7 (35)	5 (12.5)	0.02
Stopped smoking >1 year ago	2 (5.0)	11 (55)	11 (55)	18 (45.0)	<0.0001
Stopped smoking <1 year ago	22 (55.0)		2 (10)	1 (2.5)	<0.0001
Self-reported PY (years)	40 (30–45)	37 (30–43)	44 (27–52)	22 (6–37)	0.0032
NIDDM (%)	5 (12.5)	3 (15)	2 (10)	1 (2.5)	0.2
Hypertension (%)	29 (72.5)	13 (65)	16 (80)	13 (32.5)	0.0007
Previous ischemic coronary vascular disease (%)	5 (12.5)	1 (5)	4 (20)	1 (2.5)	0.2
Previous ischemic cerebral vascular disease (%)	2 (5)	0 (0)	2 (10)	1 (2.5)	0.4
Using thrombocyte 2 inhibitors (%)	38 (95)	20 (100)	18 (90)	2 (5)	<0.0001
Using cholesterol lowering medication (%)	30 (75)	15 (75)	15 (75)	7 (17.5)	<0.0001
Fontaine classification at diagnosis (%)				NA	
I	1 (2.5)	1 (5)			
IIa	28 (70)	11 (55)	17 (85)		
IIIb	9 (22.5)	6 (30)	3 (15)		
III	1 (2.5)	1 (5)			
IV	1 (2.5)	1 (5)			
Time since initial PAD-diagnosis (years)	2.2 (1.9–4.3)	4 (2–4.4)	2.1 (1.9–3.5)	NA	

*BMI, body mass index; NIDDM, non-insulin-dependent diabetes mellitus; NA, not applicable; PAD, peripheral arterial disease; IQR, interquartile range*.

*Continuous data are expressed as median with IQR. Dichotomous data are shown as *n* (%). The *p*-values were calculated using either the unpaired *t*-test with Welch’s correction when data were normally distributed or Mann–Whitney *U* test when the data were not normally distributed*.

In Table 2, we show that, in line with previous findings, median levels of the coagulation markers vWF, fibrinogen and D-dimer, were increased in the combined group of PAD patients, compared to the healthy controls. Contrasting with previous literature, PAD patients had a lower hematocrit level compared to healthy controls. Both markers of inflammation, ESR and leukocyte count, were significantly increased in PAD patients versus healthy controls; besides a significant increase in the eosinophil granulocytes, there were no significant differences in the leukocyte subpopulations.

**Table 2:** **Hematological and hemostatic parameters of the study population (median with IQR)**.

Hemostatic parameters with reference values	PAD (*n* = 40)	PAD with event during first-year FU (*n* = 20)	PAD without event during first-year FU (*n* = 20)	Healthy controls (*n* = 40)	*p*-Value
Hemoglobin (7.3–9.7 mmol/L)	8.7 (8.2–9.3)	8.6 (7.9–9.3)	8.7 (8.4–9.4)	9.0 (8.6–9.5)	0.06
Hematocrit (0.36–0.48 L/L)	0.43 (0.39–0.45)	0.44 (0.38–0.45)	0.43 (0.40–0.45)	0.44 (0.42–0.47)	0.008*
ESR (0–14 mm)	8 (6–13)	8 (7.5–13.8)	8 (5–13)	6 (3.5–7)	0.004*
Thrombocytes (130–150 × 10^9^/L)	247 (214–282)	258 (222–281)	247 (205–286)	255 (205–291)	0.62
vWF antigen (50–150%)	149 (119–174)	146 (113–159)	157 (125–203)	133 (107–154)	0.044*
RDW (11.4–14.5%)	13.0 (12.4–13.5)	13.1 (12.3–13.5)	12.8 (12.5–13.7)	12.9 (12.6–13.2)	0.66
MPV (9.2–12.7 fL)	11.1 (10.4–11.9)	11.0 (10.6–11.6)	11.2 (10.3–12.0)	10.8 (10.2–11.4)	0.31
Leukocytes (3.5–11.0 × 10^9^/L)	7.5 (6.0–9.1)	7.7 (6.3–8.9)	7.4 (6.0–9.5)	6.2 (5.6–7.5)	0.003*
Lymphocytes (15–48%)	28 (24–33)	29 (24–33)	26 (23–34)	31 (26–35)	0.21
Monocytes (4–11%)	9 (7–10)	8 (6–10)	10 (8–11)	7 (6–10)	0.06
Neutrophil granulocytes (40–70%)	60 (55–64)	60 (55–64)	60 (54–65)	58 (51–63)	0.37
Eosinophil granulocytes (0–10%)	3 (2–4)	3 (2–5)	3 (2–4)	2 (1–3)	0.012*
aPTT (23–32 s)	26 (25–27)	26 (25–27)	26 (24–28)	26 (26–27)	0.36
PT (9.9–11.5 s)	10.0 (9.8–10.2)	10.0 (9.8–10.1)	10.0 (9.8–10.3)	10.1 (9.8–10.3)	0.36
Fibrinogen (1.7–4.0 g/L)	3.6 (3.1–3.9)	3.5 (3.1–3.9)	3.6 (3.0–4.1)	3.1 (2.8–3.5)	0.015*
d-dimer (0–500 ng/mL)	528 (363–835)	497 (358–816)	564 (389–959)	366 (259–520)	0.005*

*ESR, erythrocyte sedimentation rate; vWF, von Willebrand factor; RDW, red blood cell distribution width; MPV, mean platelet volume; aPTT, activated partial thromboplastin time; PT, prothrombin time; IQR, interquartile range*.

*Values are expressed as median with IQR. The p-values between the total PAD group and the healthy control group were calculated using either the unpaired t-test with Welch’s correction during normal Gaussian distribution or Mann–Whitney U test when data were not normally distributed*.

*Significant difference (*p* < 0.05). There were no significant differences between the two PAD groups.

### Thrombin Generation in PPP

**Figure 1:**
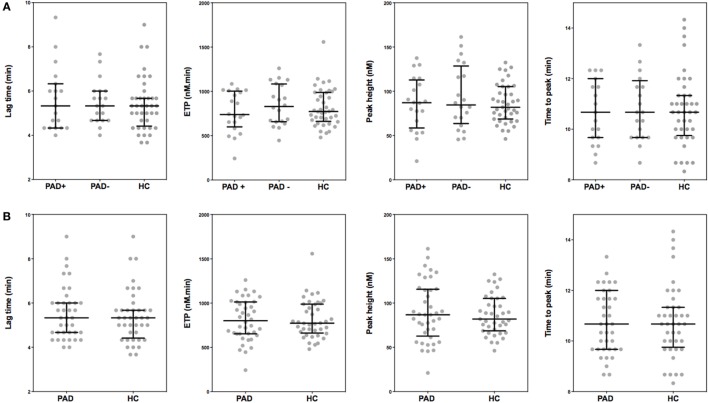
**Parameters of thrombin generation in peripheral arterial disease (PAD) patients and healthy controls**. Thrombin generation was measured following a 1 pM tissue factor trigger in the presence of 4 µM phospholipids in platelet-poor plasma of PAD patients with (PAD+) and without (PAD−) a cardiovascular event during the first year after initial diagnosis of PAD and in platelet-poor plasma of healthy controls **(A)**. Thrombin generation measurements of PAD patients both with and without event were also grouped and compared to healthy controls **(B)**. Shown are the thrombin generation parameters lag time in minutes, endogenous thrombin potential (ETP), peak height, and time to peak in minutes. Data of the groups are presented as median with interquartile ranges and were analyzed statistically as described.

Global coagulation potential was measured in PPP triggered with either 1 or 5 pM tissue factor. In Figure 1, parameters derived from the thrombin generation curve performed in PPP triggered with 1 pM tissue factor [lag time, endogenous thrombin potential (ETP), peak height (PH), and time to peak (TTP)] are depicted for both PAD patients (with and without event) and healthy controls. There are neither any significant differences between PAD patients and the healthy controls nor any significant differences between PAD patients with and without an event. Measurements performed in PPP triggered with 5 pM tissue factor showed comparable results (data not shown).

To assess possible differences in the activity of the anticoagulant protein C pathway between PAD patients and healthy controls, the same experiments were performed in the presence of 0.56 nM thrombomodulin [a concentration that was chosen such that it inhibits thrombin generation in pooled normal plasma by 50% (15)]. The addition of thrombomodulin caused a similar reduction of the ETP in both healthy controls and PAD patients.

### Thrombin Generation in WB

**Figure 2:**
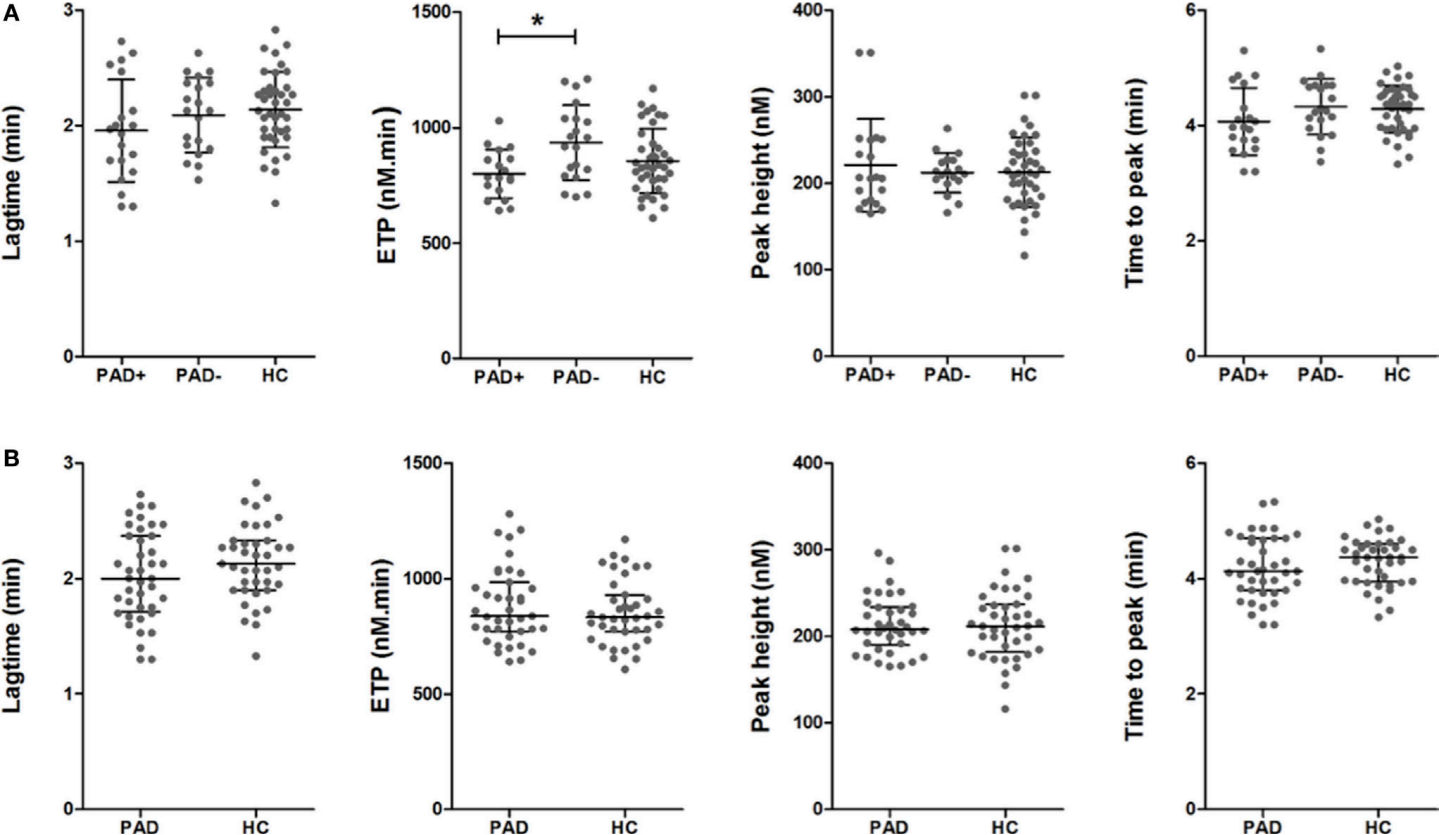
**Parameters of whole blood thrombin generation in peripheral arterial disease (PAD) patients and healthy controls**. Thrombin generation was measured following a 2.5 pM tissue factor trigger in the presence of 16.7 mM CaCl_2_ in whole blood of PAD patients with (PAD+) and without (PAD−) a cardiovascular event during the first year after initial diagnosis of PAD and in whole blood of healthy controls **(A)**. Thrombin generation measurements of PAD patients both with and without event were also grouped and compared to healthy controls **(B)**. Shown are the thrombin generation parameters lag time in minutes, endogenous thrombin potential (ETP) in nanomolars × minutes, peak height in nanomolars, and the time to peak in minutes. Data of the groups are presented as median with interquartile ranges and were analyzed statistically as described, with *p < 0.05.

In thrombin generation measurements performed in WB, thrombin generation was triggered with 0, 0.5, or 2.5 pM tissue factor and 16.7 mM CaCl_2_. In Figure 2, the parameters (LT, ETP, PH, and TTP) derived from the thrombin generation curve triggered with 2.5 pM tissue factor are depicted for PAD patients and healthy controls. We observed that the ETP values were lower in PAD patients with an event compared to PAD patients without an event [800 nM⋅min (SD = 105) versus 935 nM⋅min (SD = 163), *p* = 0.013]. We observed comparable results at 0 pM tissue factor; however, at 0.5 pM tissue factor, no significant differences were observed. There were no significant differences in any of the other thrombin generation parameters, and there were no differences between PAD patients and healthy controls.

### Rotational Thromboelastometry

**Figure 3:**
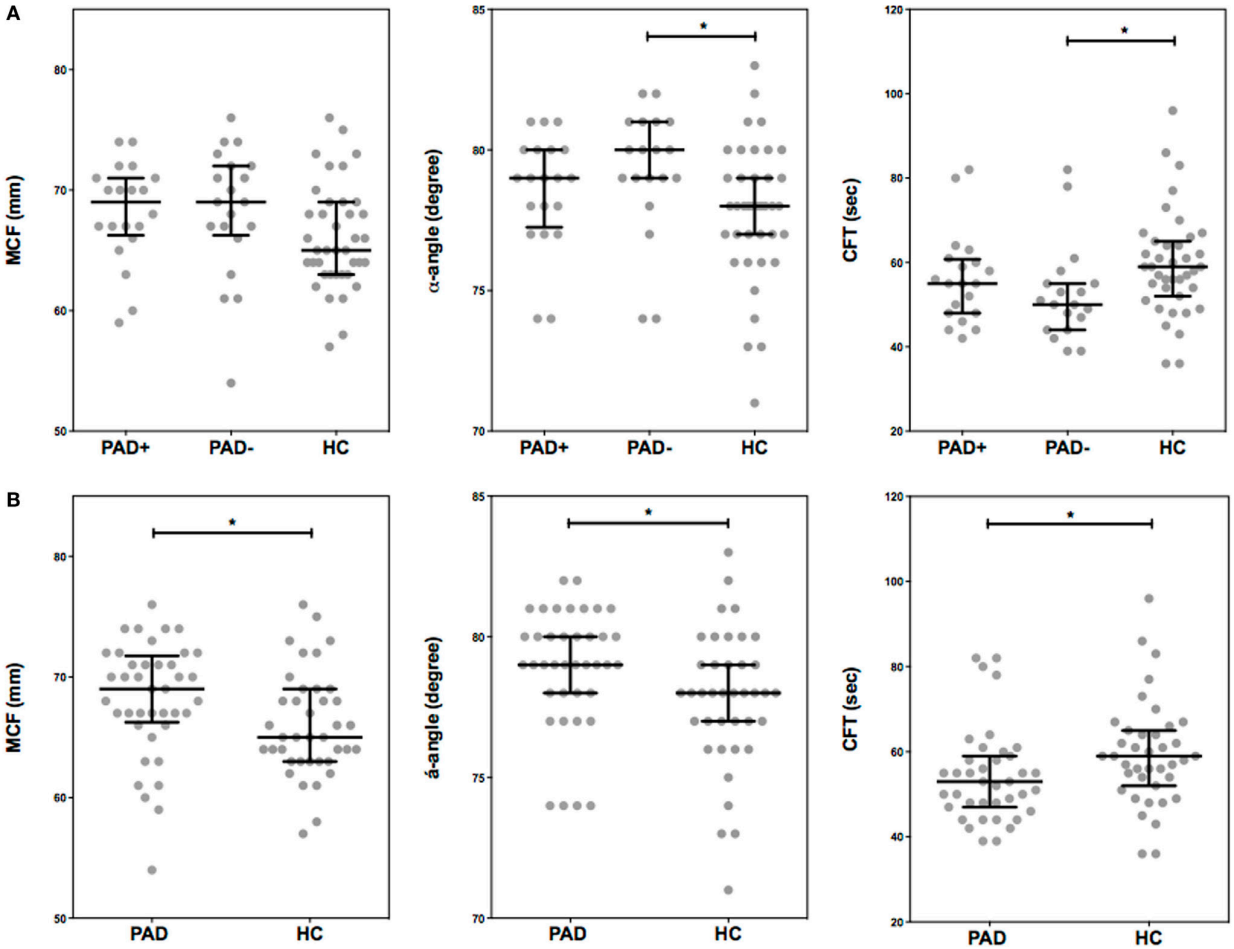
**Rotational thromboelastometry (ROTEM) (EXTEM-based ROTEM) was assessed for clot formation in whole blood of peripheral arterial disease (PAD) patients with (PAD+) and without (PAD−) a cardiovascular event during the first year after initial diagnosis of PAD and in whole blood of healthy controls (A)**. Parameters of clot formation of PAD patients both with and without event were also grouped and compared to healthy controls **(B)**. Shown are the α-angle, maximum clot firmness (MCF), and clot formation time (CFT). Data of the groups are presented as median with interquartile ranges and were analyzed statistically as described, with *p < 0.05.

To assess global clot formation, we performed WB ROTEM measurements following stimulation with 35 pM tissue factor and analyzed the different parameters (CFT, α-angle, and MCF, Figure 3). The CFT was significantly diminished in PAD patients (median CFT: PAD with event, 55 s; PAD without event, 50 s; and healthy controls, 59 s). Despite a difference of the median α-angle (denoting the speed at which a clot forms in WB) of only 1°, this difference was significant and increased in PAD patients in comparison to healthy controls (79° versus 78°, Figure 2B). This increase is, comparable to the CFT, mainly caused by the increase in PAD patients who did not experience a cardiovascular event as the median α-angle in this group was 80°. Between patients with and without event, no differences were observed.

In addition, with stable blood clot formation, the MCF, reflecting the absolute strength of the fibrin and platelet clot, was significantly increased in PAD patients versus healthy controls (median MCF: PAD, 69 mm; healthy controls, 65 mm); no differences between PAD patients with and without event were found.

To assess determinants of this increased clot formation capacity in PAD patients, we calculated the correlation coefficients of various hematological and hemostatic parameters and the ROTEM parameters of clot formation. The ESR, fibrinogen levels, and thrombocyte count were positively correlated with the parameters of clot formation. The hematocrit and hemoglobin levels on the other hand showed an inverse correlation with clot formation parameters α-angle and MCF (Table 3).

**Table 3:** **Correlation coefficients between various hematological and hemostatic parameters and the ROTEM parameters of clot formation**.

	ESR	Hemoglobin	Hematocrit	Thrombocytes	Fibrinogen
CFT	*r* = −0.5 (*p* = 3.27 × 10^−7^)	*r* = 0.5 (*p* = 3.48 × 10^−6^)	*r* = 0.5 (*p* = 1.54 × 10^−5^)	*r* = −0.5 (*p* = 6.84 × 10^−7^)	*r* = −0.6 (*p* = 1.42 × 10^−7^)
α-angle	*r* = 0.6 (*p* = 2.69 × 10^−8^)	*r* = −0.5 (*p* = 6.36 × 10^−6^)	*r* = −0.5 (*p* = 2.61 × 10^−5^)	*r* = 0.5 (*p* = 7.63 × 10^−7^)	*r* = 0.6 (*p* = 2.06 × 10^−8^)
MCF	*r* = 0.6 (*p* = 5.89 × 10^−10^)	*r* = −0.5 (*p* = 1.42 × 10^−5^)	*r* = −0.4 (*p* = 0.000365)	*r* = 0.4 (*p* = 0.000159)	*r* = 0.7 (*p* = 4.75 × 10^−13^)

*ESR, erythrocyte sedimentation rate; CFT, clot formation time; MCF, maximal clot firmness; ROTEM, rotational thromboelastometry*.

*Values calculated with the Spearman correlation coefficient*.

### tPA-Induced Fibrinolysis by ROTEM

**Figure 4:**
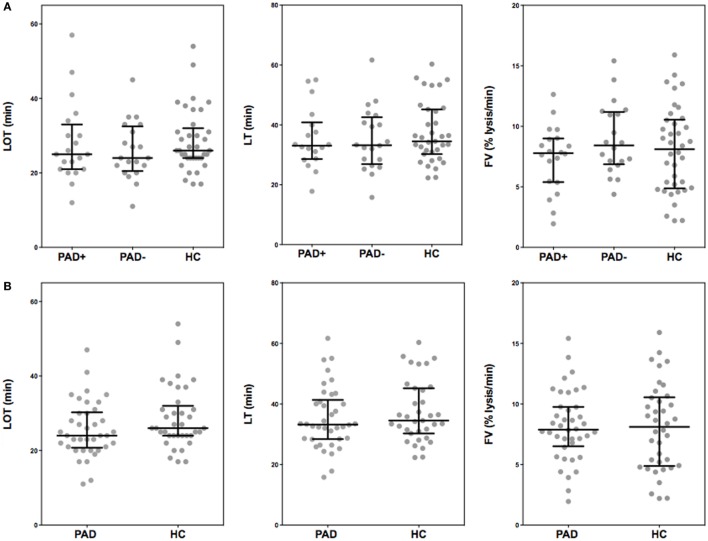
**Rotational thromboelastometry (“tPA-ROTEM”) was assessed for clot lysis in whole blood of peripheral arterial disease (PAD) patients with (PAD+) and without (PAD−) a cardiovascular event during the first year after initial diagnosis of PAD and in whole blood of healthy controls (A)**. Parameters of clot lysis of PAD patients both with and without event were also grouped and compared to healthy controls **(B)**. Shown are the lysis onset time (LOT), lysis time (LT), and the fibrinolysis velocity (FV). Data of the groups are presented as median with interquartile ranges and were analyzed statistically as described.

In Figure 4, the thromboelastometry parameters representing fibrinolysis of a blood clot in PAD patients and healthy controls are depicted. These parameters are set as the LOT (15% lysis of the clot), the lysis time (LT; when 90% clot lysis has occurred), and the FV, which is the decline of the clot between the LOT and LT in % per minute. ROTEM measurements were performed with the addition of 175 ng/mL tPA, this in order to achieve full clot lysis within 2 h. There were no significant differences between the parameters of clot lysis between PAD patients and healthy controls nor between the two PAD groups.

## Discussion

To confirm our hypothesis that patients with PAD show a hypercoagulable status, we assessed the coagulation, clot formation, and clot lysis status with a concise and state-of-the-art panel of assays. This case–control study indeed demonstrates that patients with PAD have a hypercoagulable state as indicated by increased markers of coagulation activity. However, contrary to what we hypothesized, the potential to generate thrombin upon additional stimulation with TF is not different between PAD and controls or even somewhat lower in those patients with PAD with an ischemic complication on short-term follow-up. The unaltered capacity to generate thrombin in spite of increased levels of formed thrombin suggests a hypercoagulability characterized by consumption of coagulation proteins diminishing the capacity to generate more thrombin. Such a phenomenon (lower levels of thrombin generation capacity in plasma) has recently also been suspected to be present in patients with PAD by others (16), as well as in a controlled study of cardiovascular complications in elderly subjects (17). Our study is the first to also show such an inverse association between thrombin generation, in WB, and thrombotic outcomes.

One confounding factor in these patients may be the use of statins. Several studies have indicated that the plasma thrombin generation potential significantly decreased upon the use of statins, already detectable within 2 months of starting statin therapy (14, 18–23). Such an attenuating effect on thrombin generation may play a role in the lack of difference between PAD patients and controls as 75% of our PAD patients versus only 17.5% of the healthy controls used statins to lower cholesterol levels.

The difference in WB thrombin generation must be related to the presence of cells in the WB assay. While a more pronounced level of depletion of coagulation proteins may be present in WB compared to plasma, direct effects of erythrocytes or leukocytes on thrombin generation cannot be excluded.

In the ROTEM measurements, we observed an increased ability to form a stable clot at an increased rate, without evidence of clot resistance to lysis, supporting our hypothesis of hypercoagulability in PAD patients. The α-angle and maximal clot firmness were significantly increased, whereas the CFT was significantly shortened in PAD patients, thus indicating that a clot is formed faster and has more firmness than clots formed by healthy controls. However, there were no differences in clot formation between the PAD patients who experienced a cardiovascular event and the patients who did not.

The increased ability to form a stable clot in PAD patients is most likely explained by the increased levels of fibrinogen and the increased ESR, as both were positively correlated with the parameters of clot formation (24–27). In a ROTEM-study performed by Lang et al., it was observed that the strength of a clot increased in a fibrinogen concentration-dependent matter independent of platelet count. At normal fibrinogen levels, however, the clot strength increased when platelet count increased from <10 × 10^3^ to 50–100 × 10^3^ mm^−3^. This increase in clot firmness tended to reach a plateau phase at normal platelet counts (28). The potential contribution of erythrocytes to the clot formation process requires further investigation. Previously, others observed denser and faster formed fibrin clots in patients with PAD in comparison to healthy controls (27, 29, 30). However, fibrinogen levels are not always implicated unequivocally (29). Previous research has demonstrated an increase in factor XIII, the transglutaminase that is responsible for cross-linking fibrin and thus strengthening the clot, in patients with PAD, albeit only significantly increased in women (31). This illustrates that clot formation is a complex and most likely multifactorial process in which fibrinogen cannot be assumed to be the sole player.

Increased fibrinogen levels are known to interfere with plasminogen and therefore reduce the fibrinolytic capacity (27). We therefore expected that PAD patients would have reduced fibrinolysis. However, we did not observe any differences in fibrinolysis between PAD patients and healthy controls. These findings contrast with previous findings by Undas et al. who observed that the fibrin clots of patients with PAD had a reduced susceptibility to clot lysis compared to the clots made from plasma from healthy controls. Because the latter measurements were performed in plasma, the effect of platelets and other blood cells on fibrin clot formation and lysis was not included (29).

The patients in our study are also characterized, as expected, by a chronic inflammatory state, indicated by elevations in the plasma levels of von Willebrand factor, ESR, leukocyte count, fibrinogen, and D-dimer levels. It is commonly assumed that the systemic atherosclerosis in this condition is responsible for these effects. A direct contribution of an activated coagulation system to the generation of D-dimers (due to combined fibrin formation and increased cleavage) is likely present as well (24, 32, 33).

Despite the fact that the thrombin generation potential appeared comparable between PAD patients and healthy controls, we did find a significant difference in the ability to build a stable clot between those two groups. Based on our results, the observed increase in D-dimer levels in PAD patients is not caused by increased fibrinolysis, but more likely is the consequence of the increased (inflammation related) fibrinogen levels and subsequent fibrin turnover and clot formation. Previous studies have shown a correlation between the plasma fibrinogen levels and the fibrin D-dimer levels (34–36). This was principally thought to be due to fibrin degradation products (including D-dimer) stimulating the hepatocytes to synthesize fibrinogen through an increased release of interleukin-6 (37). However, the reverse mechanism, in which increased fibrinogen contributes to hypercoagulability and increased D-dimer levels, has also been proposed (38, 39).

One of the remaining questions of our study is why we observed hardly any differences in the coagulation status between the two PAD-groups: the patients with a cardiovascular event within 1 year after PAD -diagnosis and the patients without. The fact that we did not find differences brings us to one of the limitations of this study. The defined criteria for “cardiovascular events” (being CVA, TIA, unstable AP, MI, coronary revascularization, and revascularization for PAD) may not have been set rigorously enough; a revascularization procedure for PAD for example is not necessarily a (direct) consequence of a hypercoagulable state. In hindsight, we probably should have focused more on *atherothrombotic events*, leading to acute ischemia than on *cardiovascular events*. As 17 of 20 included patients with events in our study underwent a peripheral revascularization procedure and only 3 patients experienced an atherothrombotic event, this could have influenced our outcomes. The patients who experienced an atherothrombotic event, albeit a small sample size, however, did not differ in outcomes of the various coagulation assays from the patients with a revascularization procedure. Another limitation of this study is the fact that our healthy controls did not have their ankle-brachial index measured to exclude PAD. However, we did ask the healthy control group to fill in the ECQ. This questionnaire has a high specificity (99.3%, 95% CI 98.9–100%) in excluding intermittent claudication. Therefore, eligible healthy controls that scored positive on the ECQ were excluded from study participation, thus limiting the chances of including healthy controls with PAD (40).

In conclusion, our data show clear evidence of hypercoagulability in WB as well as plasma from patients with PAD, up to the level of fibrin clot formation, without obvious effects on clot lysis. This prothrombotic state, linked to a phenotype of systemic atherosclerosis, may play an important role in the high risk of cardiovascular complications and vascular death that is typical for PAD. These findings provide additional basis for new studies on the use of anticoagulant agents, possibly in combination with antiplatelet therapy (the current cornerstone of antithrombotic management in PAD).

## Ethics Statement

Patients were included after written informed consent was obtained. The study protocol conforms to the ethical guidelines of the 1975 Declaration of Helsinki (Seoul 2008) and was approved by the local medical ethics committee (METC azM/UM).

## Author Contributions

Full access to all of the data in the study and responsibility for the integrity of the data and the accuracy of the data analysis: M-CK and HC. Study concept and design: M-CK, AC-H, and HC. Acquisition of data: M-CK and JK. Analysis and interpretation of data: M-CK, HC, and JK. Drafting of the manuscript: M-CK. Critical revision of the manuscript for important intellectual content: M-CK, JD, YH, HS, AC-H, HC, and JK, BL. Statistical analysis: M-CK and HS, and JK. Administrative, technical, or material support: JD and YH. Study supervision: M-CK, AC-H, and HC.

## Conflict of Interest Statement

The authors declare that the research was conducted in the absence of any commercial or financial relationships that could be construed as a potential conflict of interest.
